# Islet NO-Synthases, extracellular NO and glucose-stimulated insulin secretion: Possible impact of neuronal NO-Synthase on the pentose phosphate pathway

**DOI:** 10.1371/journal.pone.0315126

**Published:** 2025-01-24

**Authors:** Ingmar Lundquist, Israa Mohammed Al-Amily, Ragnar Henningsson, Albert Salehi

**Affiliations:** 1 Department of Clinical Science, SUS, Division of Islet Cell Physiology, University of Lund, Malmö, Sweden; 2 Department of Experimental Medical Science, University of Lund, Lund, Sweden; Cinvestav-IPN, MEXICO

## Abstract

The impact of islet neuronal nitric oxide synthase (nNOS) on glucose-stimulated insulin secretion (GSIS) is less understood. We investigated this issue by performing simultaneous measurements of the activity of nNOS *versus* inducible NOS (iNOS) in GSIS using isolated murine islets. Additionally, the significance of extracellular NO on GSIS was studied. Islets incubated at basal glucose showed modest nNOS but no iNOS activity. Glucose-induced concentration-response studies revealed an increase in both NOS activities in relation to secreted insulin. Culturing at high glucose increased both nNOS and iNOS activities inducing a marked decrease in GSIS in a following short-term incubation at high glucose. Culturing at half-maximal glucose showed strong iNOS expression revealed by fluorescence microscopy also in human islets. Experiments with nNOS-inhibitors revealed that GSIS was inversely related to nNOS activity, the effect of iNOS activity being negligible. The increased GSIS after blockade of nNOS was reversed by the intracellular NO-donor hydroxylamine. The enhancing effect on GSIS by nNOS inhibition was independent of membrane depolarization and most likely exerted in the pentose phosphate pathway (PPP). GSIS was markedly reduced, 50%, by glucose-6-phosphate dehydrogenase (G-6-PD) inhibition both in the absence and presence of nNOS inhibition. NO gas stimulated GSIS at low and inhibited at high NO concentrations. The stimulatory action was dependent on membrane thiol groups. In comparison, carbon monoxide (CO) exclusively potentiated GSIS. CO rather than NO stimulated islet cyclic GMP during GSIS. It is suggested that increased nNOS activity restrains GSIS, and that the alternative pathway along the PPP initially might involve as much as 50% of total GSIS. In the PPP, the acute insulin response is downregulated by a negative feedback effect executed by a marked upregulation of nNOS activity elicited from secreted insulin exciting insulin receptors at exocytotic sites of an nNOS-associated population of secretory granules.

## Introduction

Glucose-stimulated insulin secretion (GSIS) is believed to be controlled mainly by the β-cell K_ATP_ channels. Closure of these channels in response to metabolism of glucose depolarizes the β-cells resulting in activation of voltage-dependent Ca^2+^-channels followed by a rise in cytosolic [Ca^2+^]_i_, and insulin release. Additionally, intracellular stores of Ca^2+^ might be implicated [1–3]. Importantly, a rise in [Ca^2+^]_i_ also stimulates the production of NO from the neuronal constitutive Ca^2+^-dependent isoform of NO synthase *i*.*e*. nNOS (also named NOS1 or ncNOS) [4,5]. The impact of nNOS on GSIS is less understood and worth considering. We believe, according to our previous observations, that this glucose-induced stimulation of β-cell nNOS, at the initiation of the secretory process, is an important mechanism involved in the modulation of GSIS, where nNOS can act as a strong negative feedback regulator, primarily to avoid acute hypersecretion of the hormone [6]. Moreover, previous observations showing an abnormally enhanced and persisting nNOS activity in the islets of the young Goto-Kakizaki (GK) rat, a nonobese model of Type 2 Diabetes (T2D), suggested a possible involvement of nNOS also in the development of T2D [7,8]. Another NOS activity, the Ca^2+^ calmodulin-independent and inducible isoform of NOS (iNOS), also named NOS2, seems to have no immediate influence on the secretory process, since it is usually expressed first after being induced by certain stressful stimuli including various immunogenic or inflammatory processes [9,10]. However, iNOS can also be induced by non-immunogenic stimuli such as persistent hyperglycemia as e.g. in the young GK rat, or in human T2D [7,8,11]. Hence, iNOS was found to be expressed and activated in the β-cell not only in the autoimmune Type 1 Diabetes (T1D) [9,10], but also in connection with longstanding hyperglycemia, often referred to as glucotoxicity [11,12]. This is important, since iNOS-derived NO is inclined to damage the β-cell in the long run [9,10], while the importance and site of action of both acute and extended changes in nNOS activity are less understood. Notably, a differential action between nNOS and iNOS might also have a morphological background, since nNOS is mainly associated with a large population of the insulin secretory granules and to a lesser extent with the mitochondria [13], whereas iNOS seems to be uniformly distributed in the β-cell cytoplasm [14]. Hence, nNOS and iNOS evidently display distinct spatiotemporal characteristics that might be of great importance for β-cell function, and dysregulation of NOS activities seems capable of playing an important role in the pathophysiology of T2D. Such a notion was underlined by the finding that postmortem isolated human islets from a T2D population displayed a highly increased expression level of both nNOS and iNOS compared to controls [15].

Thus, in view of the present profound confusion with regard to our knowledge of the involvement of nNOS in islet physiology and pathophysiology, the aim of the present investigation was to study in more detail, in islets from healthy mice, the basal function of islet nNOS versus iNOS in GSIS, emphasizing simultaneous measurements of these isoforms to elucidate in particular the mode of action of nNOS and its final effects on the secretory machinery. A second aim was to elucidate whether changes of extracellular NO might influence the effect of β-cell nNOS on GSIS. In addition, the effect of extracellular NO was compared to another gaseous messenger molecule i.e. carbon monoxide (CO).

## Materials and methods

### Drugs and chemicals

Fatty acid free bovine serum albumin (BSA) and fatty acid free fetal bovine serum albumin (FSA) were from Boehringer Mannheim, Germany. NG-nitro-L-arginine methyl ester (L-NAME), NG-monomethyl-L-arginine (L-NMMA), dehydroepiandrosterone (DHEA), diazoxide, hydroxylamine (H_3_NO), dimethylsulfoxide (DMSO), 4-acetamido-4-isothiocyanostilbene-2,2´-disulfonic acid (SITS) and all other chemicals were from Merck AG, (Darmstadt, Germany) or Sigma-Aldrich (St. Louis, MD, USA) unless otherwise stated. The radioimmunoassay kits for insulin determination were from Millipore, USA.

### Animals

Female mice of the Naval Medical Research Institute (NMRI) strain, weighing 25–35 g, were used throughout the experiments. They were given a standard pellet diet (B&K) and tap water ad libitum. The experimental procedures including the method of sacrifice (cervical dislocation) were approved by the Ethics Committee for Animal Research at the University of Lund, Lund, Sweden (Dnr 1057/2020).

### Source of human pancreatic islets

Isolated human pancreatic islets from non-diabetic cadaveric female donors were from Prodo Laboratories INC (USA). The islets were hand-picked under stereomicroscope prior to use. All the experiments performed in the current study were compiled with ethical regulation and approved by the local Ethical Committees at Lund University, Sweden.

### In vitro insulin release studies

The general procedures for studying *in vitro* insulin secretion has been described previously [6,16]. Preparation of isolated pancreatic islets from the mouse was performed by retrograde injection of a collagenase solution via the bile-pancreatic duct [17]. In batch incubation experiments, freshly isolated islets were preincubated for 30 min at 37 C in Krebs–Ringer bicarbonate (KRB) buffer, containing (in mM): NaCl (120), KCl (4.7), CaCl_2_ (2.5), KH_2_PO_4_ (1.2), MgSO_4_ (1.2), HEPES (10), NaHCO_3_ (25) and 1 mM glucose. Then the pH was adjusted to 7.4 and the buffer was supplemented with 0.1% bovine serum albumin. Each incubation vial was gassed with 95% O_2_ and 5% CO_2_ to obtain constant pH and oxygenation. After preincubation, the buffer was changed to a fresh KRB buffer supplemented with the different test agents, and the islets (12 islets per 1.0 ml of medium in each incubation vial, or 50 islets /vial (for cyclic nucleotide measurements), were incubated for 60 min. All incubations were performed at 37° C in an incubation box (30 cycles per min). Immediately after incubation aliquots of the medium were removed and frozen for subsequent assay of insulin. In culture experiments, the islets were cultured for 24 h in RPMI-1640 (SVA, Uppsala, Sweden) containing 5, 11.1, or 20 mM glucose supplemented with 10% calf serum, 100 U/ml penicillin and 10 μg/ml streptomycin. After culture and washing, the islets were preincubated and incubated as described above. In experiments with DHEA the drug was dissolved in DMSO to a final concentration of 100 μM DHEA in 0.01% DMSO. Similarly, in experiments with SITS, the drug was dissolved in DMSO.

### Assay of islet NOS activities

Preincubation and incubation of freshly isolated islets were performed as stated above with the exception that each incubation vial contained 250 islets in 1.5 ml of buffer solution. After an incubation period of 60 min, aliquots of the medium were removed for determination of insulin. Thereafter, the islets were washed and collected in 840 μl of buffer, containing 20 mM HEPES, 0.5 mM EDTA and 1.0 mM D,L-dithiothreitol and stored at -20 C. On the day of the assay, the islets were sonicated on ice and for measuring nNOS activity the buffer solution was enriched with 0.45 mM CaCl_2_, 2 mM NADPH, 25 U/ml calmodulin and 0.2 mM L-arginine in a total volume of 1 ml. For the determination of iNOS activity, which is Ca^2+^ and calmodulin independent both Ca^2+^ and calmodulin were omitted from the buffer. The homogenate solution was then incubated at 37° C under constant air bubbling, 1.0 ml/min for 2 h. Aliquots of the incubated homogenate (200 μl) were then passed through an 1-ml Amprep CBA cation exchange column for determination of L-citrulline by high-performance liquid chromatography (HPLC). This analytical technique is used to separate, identify or quantify each component in a mixture. The mixture is separated using the principle of column chromatography and then identified and quantified by spectroscopy. The method has been described in detail [6,16]. As L-citrulline and NO are generated in equimolar amounts, and as L-citrulline is stable, whereas NO is not, L-citrulline is the preferred parameter when measuring NO production. The activity unit was determined as the formation of L-citrulline pmol/mg protein/min (incubation time). Protein was determined according to Bradford [18] on samples from the original homogenate.

### Effects of exogenously administered NO and CO

One hundred ml of the KRB buffer containing (in mM): NaCl (120), KCl (4.7), CaCl_2_ (2.5), KH_2_PO_4_ (1.2), MgSO_4_ (1.2), HEPES (10), NaHCO_3_ (25), 1 mM glucose with pH adjusted to 7.4 was purged of O_2_ by helium and saturated with either NO or CO, whereupon it was supplemented with 0.1% bovine serum albumin. Control buffer was saturated with helium. The solubility of CO (2.3 ml/100 ml H_2_O) is very close to the solubility of NO (4.6 ml/100 ml H_2_O). After dilution islets were then incubated in these media as described above, except that the incubation vials were gassed with air instead of 95% O_2_/5%CO_2_ [19,20].

### Measurement of cyclic GMP and cyclic AMP

After incubation the islets were washed and stored in 500 μl of ice-cold 10% trichloroacetic acid (TCA) containing the phosphodiesterase inhibitor IBMX (200 μM) followed by immediate freezing in a -70% ethanol bath. Before assay, 500 μl of H_2_O was added, and the samples were sonicated for 3 x 5 sec followed by centrifugation at 1100 g for 15 min. The supernatants were collected and extracted with 4 x 2 ml of water-saturated diethyl ether. The aqueous phase was removed and freeze-dried using Lyovac GT 2 freeze- drier. The residue was then dissolved in 450 microliter of 50 mM sodium acetate buffer (pH 6.2). The amounts of cyclic GMP and cyclic AMP were quantified with ^125^I-labeled cyclic GMP ^125^I-labeled cyclic AMP RIA kit (RIANEN; Du Pont, Boston, MA). (^3^H)cyclic GMP or (^3^H)cyclic AMP was added to the TCA homogenate to determine the recovery of cyclic GMP and cyclic AMP during the ether extraction, [20]. The mean recovery was 85%.

### Immunofluorescence and confocal microscopy

After 24h culture at 5 or 11.1 mM glucose, the islets were washed, and preincubated for 30 min at 5 mM glucose. The islets were then washed again and fixed with 3% paraformaldehyde for 10 min, followed by permeabilization with 0.1% Triton X-100 for 15 min. Unspecific sites were blocked with 5% normal donkey serum (Jackson Immunoresearch Laboratories Inc., West Grove, PA, USA) in PBS. Polyclonal anti-iNOS antibody (StressGen Biotechnologies Corp., Victoria, BC, Canada) (1:100) in combination with Cy2-conjugated anti-rabbit IgG (Jackson Immunoresearch Laboratories Inc.) (1:150) and a guinea pig-raised anti-insulin antibody (Eurodiagnostica, Malmö, Sweden) (1:1000) followed by incubation with a Cy5-conjugated anti-guinea pig IgG antibody (Jackson Immunoresearch Laboratories Inc.) (1:150) for staining of iNOS or insulin were used. The fluorescence was visualized with a Zeiss LSM510 confocal microscope (Carl Zeiss Inc., Jena, Germany) by sequentially scanning at (excitation/emission) 488/ 505–530 nm (Cy2) and 633/>650 nm (Cy5). For scoring of iNOS-positive cells in islet tissue, multiple fields for each section were analyzed under blind conditions. The mean fluorescence intensity of cellular iNOS was analyzed using Zeiss LSM5 analysis software. All fluorescence intensity measurements were performed with randomly selected islets from six mice or three humans (because of limited access to human islets).

### Statistics

The data are presented as mean±SEM. The statistical differences between groups were determined by analysis of variance followed by Tukey-Kramer’s multiple comparisons test or Student’s t-test where applicable.

## Results

### Basal characteristics of the islet NOS-NO system in GSIS

**Fig 1:**
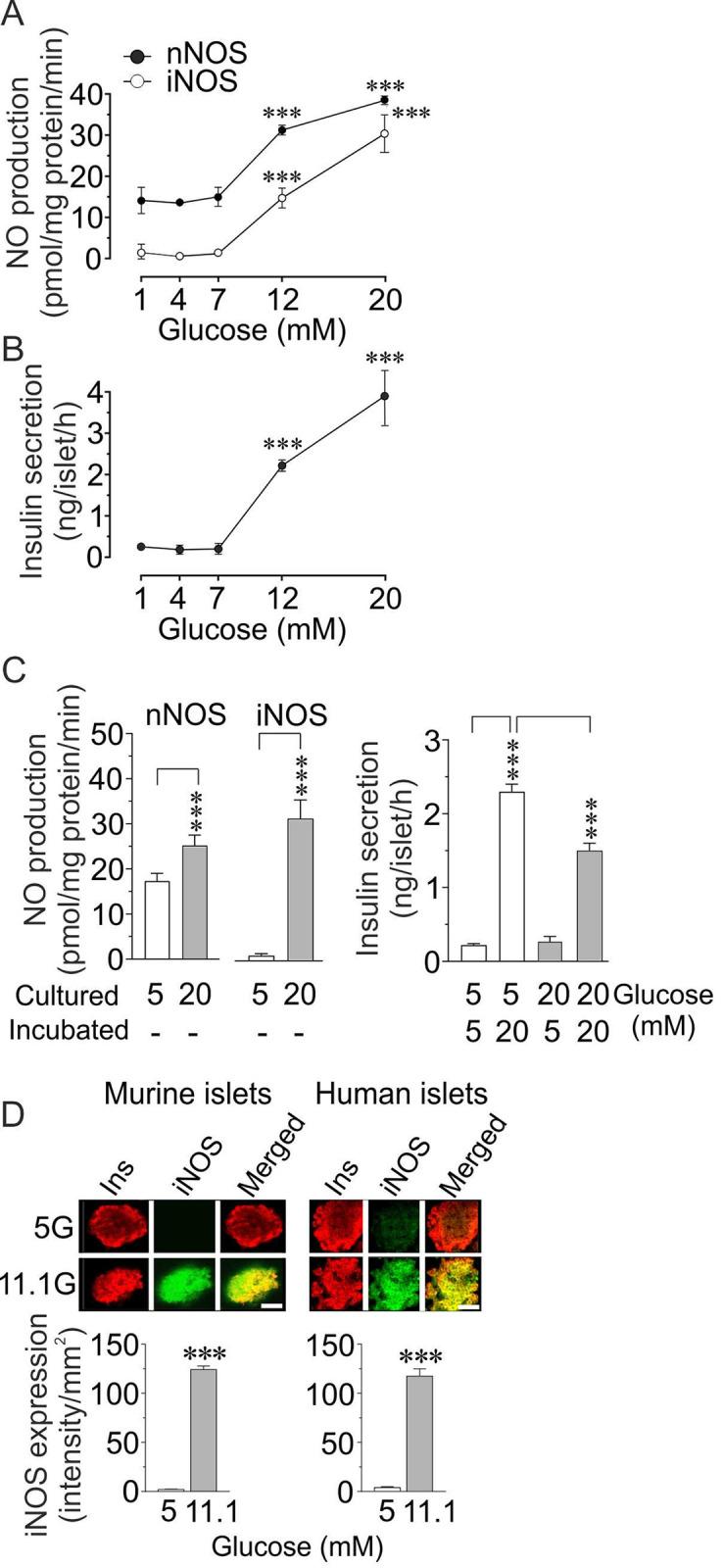
**(A, B) Basal characteristics of the relationship between nNOS, iNOS and GSIS.** Concentration-response relationship for glucose concentration and the activities of nNOS and iNOS (A) as well as insulin secretion (B) in murine islets incubated for 60 min. Means±SEM for 3–7 batches of islets pooled from 10 mice. Asterisks (*) denote significant difference versus 7 mM glucose. ***p<0.001. **(C)** Activities of nNOS and iNOS in murine islets cultured at 5 or 20 mM glucose for 24h. Isolated islets were cultured at 5 or 20 mM glucose and enzyme activities for nNOS and iNOS were measured. In a parallel experiment with similar conditions (culture at 5 and 20 mM glucose), the islets were washed and incubated for 60 min in buffer solutions with 5 or 20 mM glucose, and insulin secretion was measured. Means±SEM for n = 4–6 batches of islets in each group performed at different occasions. ***p<0.001. **(D)** Confocal microscopy and fluorescence intensity measurements of iNOS expression levels in murine and human islets, respectively, cultured for 24h at 5 or 11.1 mM glucose. A representative confocal image of the islets double-immunolabelled for insulin and iNOS protein. Insulin staining appears as red and iNOS as green staining, respectively. Co-localization of insulin/iNOS is seen as an orange-yellowish fluorescence. Means±SEM are shown for n = 6–8 (4 mice) and n = 3 (human) experimental observations in each group performed at different occasions. ***p<0.001. Scale bars indicate 20 um. Bar graphs in the lower part of image represent mean fluorescence intensity of iNOS quantified per mm^2^ using Zen 2009 software in 6–8 images per mouse or human in each group, in the islets cultured at 5 mM or 11.1 mM glucose.

The pattern of nNOS and iNOS activities in relation to basal and glucose-induced insulin secretion in isolated murine islets after a conventional 60 min. incubation period is shown in Fig 1A and 1B. The activity of nNOS was low and stable at basal glucose levels (1–7 mM). No iNOS activity could be detected. Basal insulin levels were also low and stable. After raising the glucose concentrations both nNOS and iNOS activities were increased along with an increasing insulin secretion thus giving no indication of whether nNOS or iNOS or both might be implicated in the secretory process. Notably, during islet culturing or preincubation glucose levels above basal are often used before the islets are subjected to acute GSIS experiments and might thus influence the results. Here we show (Fig 1C) that culturing murine islets for 24h at high, 20 mM glucose, as compared to culturing at basal, 5 mM glucose, induced a strong increase in NO evolution originating in particular from iNOS activity. After washing, a subsequent 60 min. incubation period at high glucose with these islets revealed a highly significant inhibitory effect on GSIS as compared to control islets cultured at 5 mM basal glucose.

#### Human islets

Moreover, to investigate whether human islets would react in a similar manner as murine islets after high glucose culturing we compared the influence of glucose on iNOS expression in murine and human islets by measuring iNOS fluorescence intensity levels. Fig 1D shows that both murine and human islets cultured for 24h in regular RPMI 1640 medium containing 11.1 mM glucose (approximately a half-maximal glucose concentration; Fig 1B) displayed very high and similar expression levels of iNOS protein. In islets cultured at 5 mM glucose no iNOS expression was detectable. Hence, culturing or preincubating islets at glucose concentrations above basal levels will induce a persistent and extremely high iNOS expression and activity and confuse the interpretation of subsequent measurements of nNOS activity and NO generation in GSIS and thus also the interpretation of the effects of nNOS activity in downstream experiments of the insulin secretory process.

### Importance of differentiating between nNOS and iNOS activities impacting GSIS. The role of nNOS in the pentose phosphate pathway

**Fig 2:**
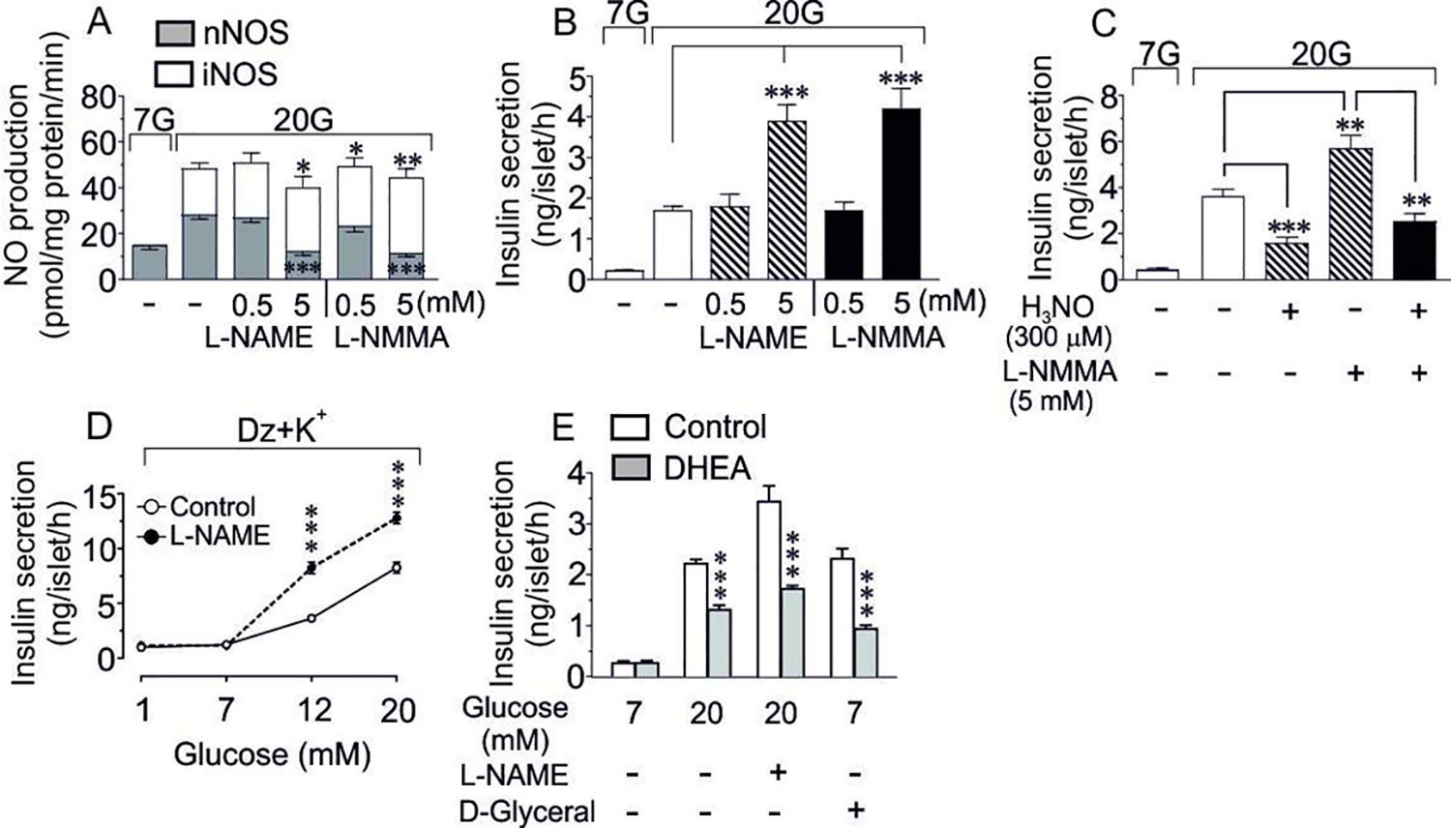
**(A, B) Differentiating between nNOS and iNOS activities impacting GSIS in isolated murine islets.** Effect of a low, 0.5 mM or a high, 5 mM, concentration of the selective nNOS inhibitors L-NAME and L-NMMA on nNOS and iNOS activities (A) as well as insulin secretion at 20 mM glucose (B) in isolated murine islets. 7 mM glucose (7G) was used as a basal control. Means±SEM for 4–6 batches of islets in each group are shown. *p<0.05, **p<0.01 and ***p<0.001. **(C)** Effect of the intracellular NO donor hydroxylamine (300 μM) on GSIS (20 mM) in the absence and presence of the selective nNOS inhibitor L-NMMA (5 mM). 7 mM glucose (7G) was used as a basal control. Means±SEM for 5–8 batches of islets in each group are shown. **p<0.01 and ***p<0.001. **(D)** Concentration-response relationship for glucose concentration and insulin secretion in the absence or presence of 5 mM L-NAME in an incubation buffer provided with 250 μM diazoxide (to keep the ATP-sensitive K^+^-channels open) and 30 mM K^+^ to depolarize the β-cells. Means±SEM for 6 batches of islets in each group performed at six different occasions are shown. ***P<0.001. **(E)** Insulin secretion at 7 or 20 mM glucose in the absence or presence of the glucose-6-phosphate dehydrogenase inhibitor DHEA (100 μM). Addition of 5 mM L-NAME or 10 mM D-glyceraldehyde as indicated. 7 mM glucose (7G) was used as a basal control. Means±SEM of 7–9 experiments in each group are shown. ***p<0.001.

To elucidate the relative contribution of nNOS *vs* iNOS activity influencing GSIS we compared the effect of two well-characterized NOS-inhibitors i.e. L-NAME and L-NMMA, previously shown to be useful for nNOS inhibitors in isolated islets [6,21]. Fig 2A and 2B shows that the pattern of nNOS-derived NO production during GSIS (20 mM glucose) was inversely related to the pattern of insulin release, the insulin secretory activity being highly increased when the nNOS activity was strongly suppressed. In contrast, the iNOS activity was modestly increased. Hence, a late [6] and significant increase in iNOS-derived NO production might give a false impression of iNOS-derived NO being a positive modulator of GSIS when no discriminating measurement between nNOS vs iNOS activity is performed. A lower concentration (0.5 mM) of the nNOS-inhibitors did not influence either nNOS activity or insulin secretion. However, iNOS activity was modestly increased by the lower dose of L-NMMA without affecting insulin secretion (Fig 2A and 2B). In these experiments 7 mM glucose was used as basal control.

#### NO donor hydroxylamine

To add more to our finding that an inhibitory effect on GSIS might be created by an increased nNOS-derived NO generation inside the β-cell, we performed a mimicking experiment with the intracellular NO donor hydroxylamine (H_3_NO) to elucidate if intracellularly released NO could reverse the enhanced insulin-releasing effect induced by the nNOS inhibitor L-NMMA in the presence of high (20 mM) glucose. Fig 2C shows that hydroxylamine induced a substantial reduction of GSIS. 7 mM glucose was used as basal control in this experiment. Moreover, the augmented insulin release induced by a combination of high glucose + L-NMMA was likewise suppressed back by hydroxylamine to the same level as recorded with high glucose + hydroxylamine, emphasizing that intracellular NO production, as mimicked by hydroxylamine-generated NO, is able to inhibit GSIS (Fig 2C).

#### Membrane depolarization and nNOS activity

To elucidate whether the intracellular action of nNOS as a negative modulator of GSIS might be directly dependent on membrane depolarization events, we then performed a concentration-response study with K^+^-diazoxide-treated islets in the absence and presence of L-NAME. Fig 2D shows that addition of L-NAME to such islets had no effect on insulin secretion at basal glucose (1–7 mM glucose), while inducing a strong amplification of GSIS at 12 and 20 mM of glucose, suggesting that the main inhibitory effect of intracellular nNOS-derived NO on GSIS is operating largely independent of membrane depolarization events. Hence, the negative modulation by nNOS on GSIS seemed less likely to affect primarily the glycolytic and the mitochondrial triggering effects of glucose.

#### The pentose phosphate pathway (PPP)

Instead, our attention was directed to the pentose phosphate pathway (PPP) and the close morphological association between nNOS and a large population of insulin secretory granules [13,22]. The production of NO from nNOS is an early event in GSIS [23] coinciding with the initiation of glucose metabolism via the PPP, since glucose-6-phosphate dehydrogenase (G-6-PD) produces cytosolic NADPH, being a prerequisite for both the induction of nNOS activity and also for the insulin secretory pathway along the PPP [4,5,24]. Moreover. NADPH is also a key factor for the insulin secretory process transduced by the cytosolic glutaredoxin-1, a redox protein being preferably associated with the secretory granules [25,26]. Hence, there is room for an important competition for cytosolic NADPH between nNOS and the NADPH-dependent glutaredoxin-1 system implicating either a negative modulation of GSIS, where nNOS utilizes NADPH and produces NO, or a positive modulation, where an nNOS-inhibitor, e.g. L-NMMA or L-NAME, is present and the NADPH-driven glutaredoxin-1 system is activated. Notably, L-NMMA as well as ADMA (Assymetric Di-Methyl-Arginine), another nNOS inhibitor, occur endogenously in the circulation [22]. Fig 2E shows that the insulin response to high glucose (20 mM) was reduced by approximately 50% by the recognized G-6-PD inhibitor dihydroxyepiandrosterone (DHEA) [24] suggesting the PPP being responsible for approximately half of the total GSIS response. Basal control level of glucose was 7 mM. Further, when L-NAME was added to high glucose the insulin response was almost doubled and addition of DHEA to the glucose + L-NAME combination resulted again in a similar reduction of the total response (Fig 2E). Finally, in an attempt to “separate” the glucose effect on the PPP from the glycolytic and the mitochondrial pathways, we added a mixture of a high insulin-secretory concentration of D-glyceraldehyde (10 mM) [27] and a basal, substimulatory glucose concentration (7 mM) to the incubation medium (Fig 2E). D-glyceraldehyde is considered a functional, if not ideal, analogue of glucose below the triose phosphate level, being able to give approximately the same yield of ATP as high glucose [28]. Hence, a maximal secretagogue concentration of D-glyceraldehyde [27] together with a basal, nontriggering concentration of glucose would then be expected to possibly recapitulate a “true” effect, if any, on PPP-metabolized glucose. Fig 2E shows that this combination induced a similar insulin response as high glucose, and that the inhibitory effect of DHEA was still very pronounced. These data might thus suggest that the cytosolic NADPH emanating from G-6-PD activity is an exclusive source for competition between nNOS and the NADPH-glutaredoxin-1 system enabling increased nNOS activity to negatively regulate the PPP-stimulated insulin response.

### Possible relevance of an extracellular approach of NO on GSIS

**Fig 3:**
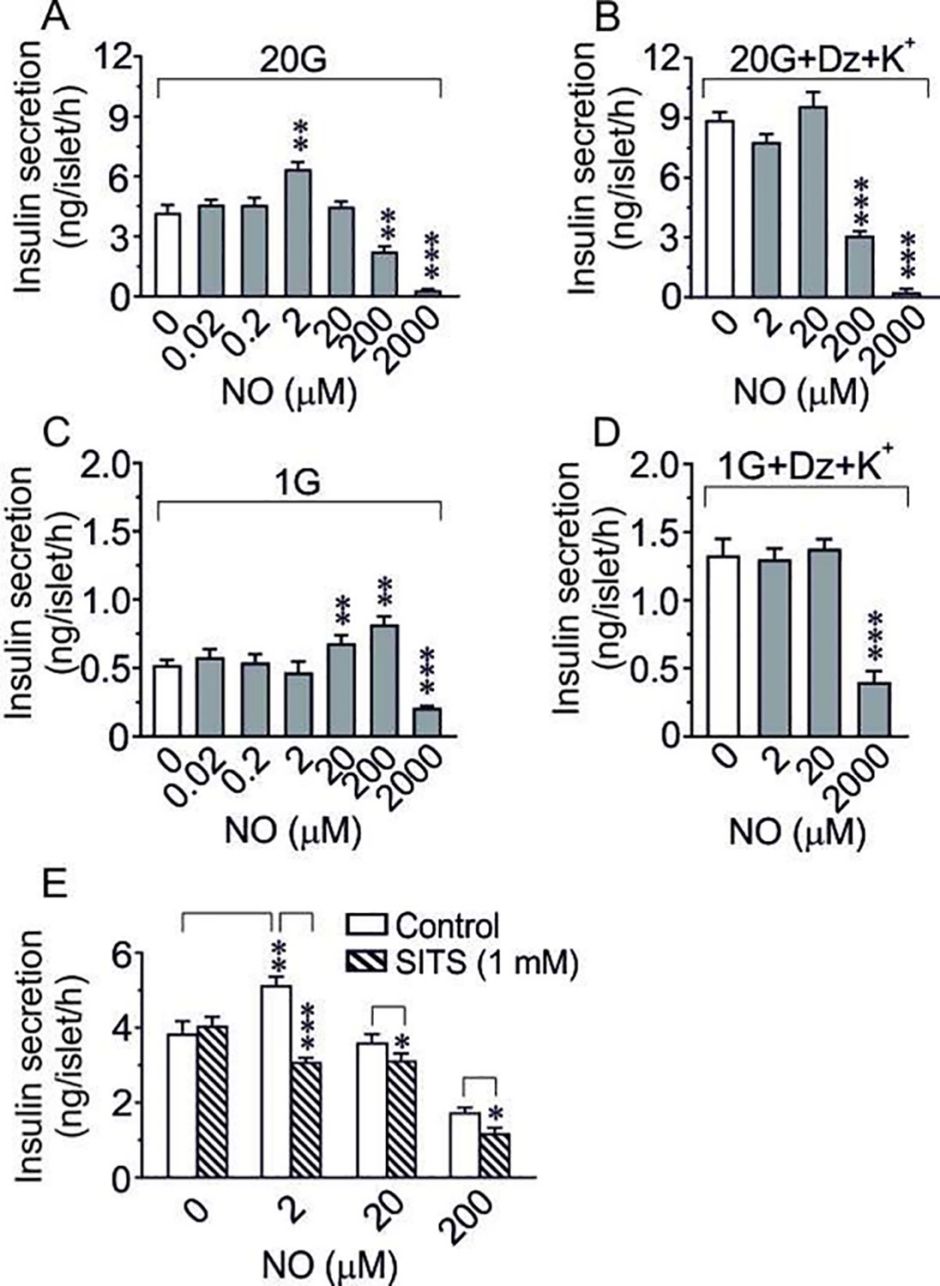
**(A, B) Concentration-response effects of NO gas on GSIS.** Murine islets were incubated at 20 mM glucose in the absence (A) or presence (B) of 250 μM diazoxide+30 mM K^+^. The different NO concentrations were prepared by diluting a buffer solution saturated with NO (2 mM). Means±SEM of 7–8 batches of islets in each group are shown. **p<0.01, ***p<0.001. **(C, D) Concentration-response effects of NO gas on basal insulin secretion.** Murine islets were incubated at 1 mM glucose in the absence (C) or presence (D) of 250 μM diazoxide+30 mM K^+^. The different NO concentrations were prepared by diluting a buffer solution saturated with NO (2 mM). Means±SEM of 6–8 batches of islets in each group are shown. **p<0.01, ***p<0.001. **(E) Effect of different concentrations of NO gas on GSIS in the absence or presence of the -SH blocker SITS.** (E) Murine islets were incubated at 20 mM glucose in the absence (control) or presence of the–SH blocker SITS (1 mM). The different NO concentrations were prepared by diluting a buffer solution saturated with NO (2 mM). Means±SEM of 7–8 batches of islets in each group are shown. *p<0.05, **p<0.01 and ***p<0.001.

To test whether the liberated NO might have an extracellular influence on GSIS, a series of experiments with addition of different concentrations of pure NO gas to isolated islets was performed. NO is an unstable and very reactive molecule, and several previous studies using exogenous administration of various NO donors or NO gas to isolated islets, isolated β-cells, or different β-cell lines suggested extracellularly donated NO being either inhibitory or stimulatory to GSIS [6,29]. Here we performed a concentration-response experiment with administration of pure NO gas to isolated islets at both high and low glucose. Fig 3A shows that NO gas has a dual action on GSIS. No effect was recorded at low levels of NO (0.02–0.2 μM), a significant increase was noted at 2 μM, no effect at 20 μM, while higher concentrations gradually suppressed GSIS down to 10% of the controls at 2 mM of NO (saturated concentration). To test for a possible membrane effect we used depolarized islets (high K^+^ and diazoxide). Fig 3B shows that the stimulatory effect of NO was abolished, while the inhibitory effect at higher concentrations of NO, if anything, was still more pronounced in depolarized islets (Fig 3B). Interestingly, at a basal glucose concentration (1 mM) the administration of exogenous NO at concentrations of 20 and 200 μM, induced a marked increase in insulin release. Saturated NO, however, was still strongly inhibitory (Fig 3C). Notably, the stimulatory, but not the inhibitory, effect of exogenous NO was abolished in depolarized islets at basal glucose (Fig 3D). Since NO is known to affect the activity of numerous regulatory proteins in cells and cell membranes by S-nitrosylation processes [30,31] we speculated that the stimulatory effect of low concentrations of exogenous NO might be exerted by activating membrane thiols also on the β-cells. Thus we performed a series of experiments with SITS, a compound known to avidly bind to the plasma membrane and thereby blocking important thiol-groups [32]. Fig 3E shows that SITS by itself did not influence GSIS. However, application of SITS converted the stimulatory action of NO at 2 μM to a highly significant inhibitory effect, and the absence of an effect at 20 μM NO in untreated islets was now inhibitory in the presence of SITS. Also, the suppressive effect at 200 μM NO was still significant. Hence, overall these data suggested a different mode of action of exogenous NO vs intra-β-cell NO on the insulin secretory machinery, exogenous NO at certain concentrations being able to stimulate insulin release, preferentially so at low glucose.

#### Carbon monoxide (CO), NO and cyclic nucleotides

**Fig 4:**
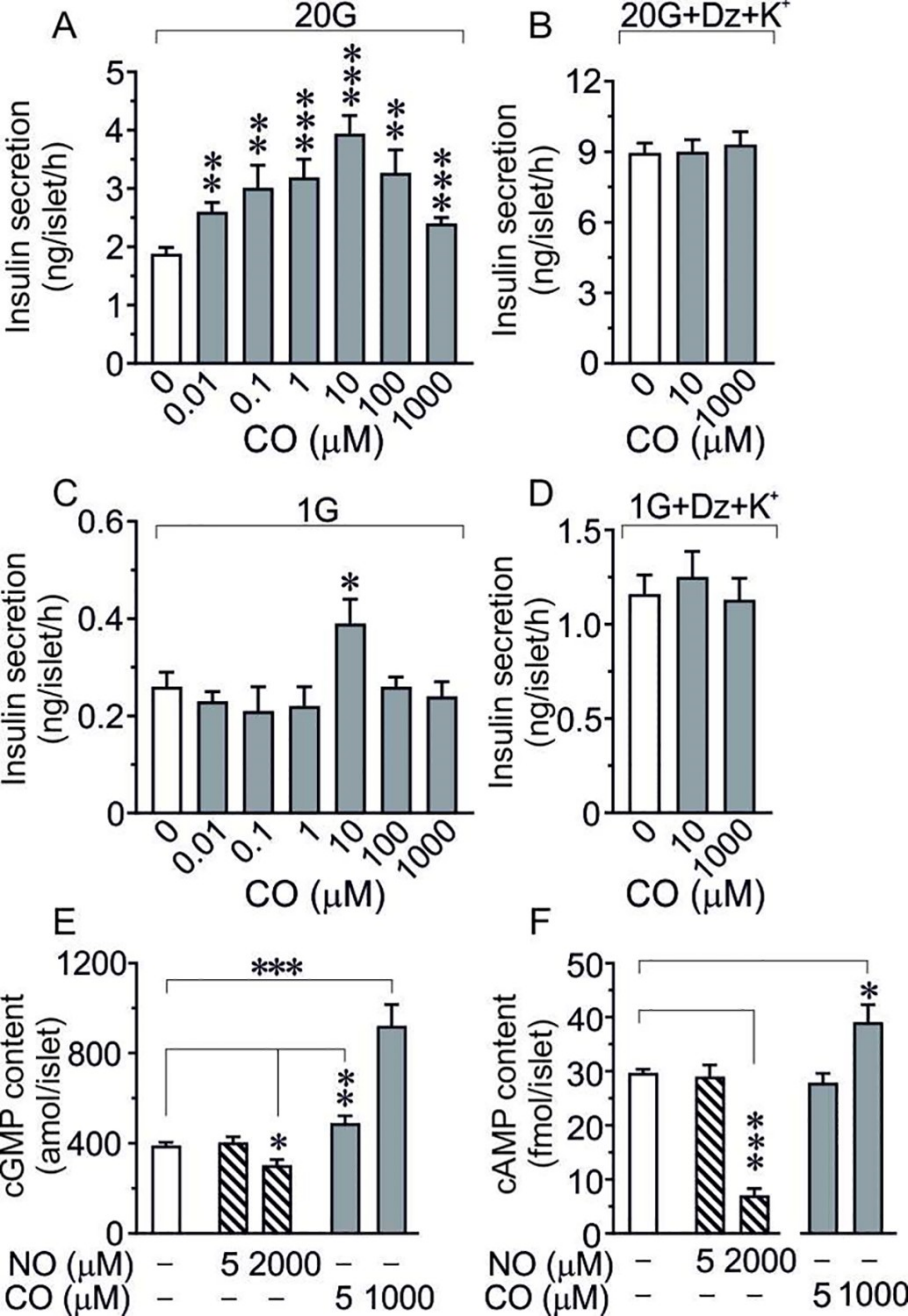
**(A, B) Concentration-response effects of CO gas on GSIS.** Murine islets were incubated at 20 mM glucose in the absence (A) or presence (B) of 250 μM diazoxide+30 mM K^+^. The different CO concentrations were prepared by diluting a buffer solution saturated with CO (1 mM). Means±SEM of 8–16 (A) or 6–8 (B) batches of islets in each group are shown. **p<0.01, ***p<0.001. **(C, D) Concentration-response effects of CO gas on basal insulin secretion.** Murine islets were incubated at 1 mM glucose in the absence (C) or presence (D) of 250 μM diazoxide+30 mM K^+^. The different CO concentrations were prepared by diluting a buffer solution saturated with CO (1 mM). Means±SEM of 7–8 batches of islets in each group are shown. *p<0.05. **(E, F) Influence of a low and a high dose of NO or CO gas on the accumulation of cyclic GMP (E) or cyclic AMP (F)** in GSIS at high glucose (20 mM) in isolated murine islets. Cyclic GMP is expressed as amol/islet and cyclic AMP as fmol/islet. Means±SEM of 4–11 batches of islets in each group. *p<0.05, **p<0.01, ***p<0.001.

The complex pattern of GSIS following addition of NO gas to isolated islets encouraged us to include a comparative experiment using another gaseous transmitter, carbon monoxide (CO). Previous studies have shown the presence of a constitutive CO-producing enzyme, heme oxygenase-2 (HO-2), in islet β-cells [19,33,34]. Moreover, the HO-substrate hemin, as well as CO gas, were found to markedly increase GSIS [19]. Fig 4A and 4B shows the pattern of GSIS at high glucose in the presence of increasing concentrations of exogenous CO. It is seen that GSIS was extremely sensitive to exogenous CO, amplifying the insulin response to glucose in a concentration as low as 0.01 μM. Although the concentration-response curve was bell-shaped a saturated concentration of CO still augmented the GSIS by 40% (Fig 4A). In depolarized islets, however, the impact of CO was totally abolished (Fig 4B) suggesting that, in contrast to NO, the effect of CO involved membrane depolarization events. Moreover, there was only a slight positive effect of CO (10 μM) at basal glucose and no effect in depolarized islets (Fig 4C and 4D). Since NO donors and extracellular NO gas, as well as CO gas, are known to stimulate the formation of cyclic GMP in the β-cell [19,20,29], we finally performed a series of experiments with NO and CO in both a low and a high concentration and measured cyclic GMP as well as cyclic AMP levels during GSIS at high glucose. Fig 4E shows that a low dose of NO had no effect on either cyclic GMP or cyclic AMP at high glucose, while a high dose of NO induced an inhibitory action on both cyclic nucleotides, especially on cyclic AMP. Low dose CO had no effect on cyclic AMP but stimulated cyclic GMP. High dose CO stimulated the formation of both cyclic GMP and cyclic AMP. Hence, CO rather than NO stimulated the cyclic GMP system and GSIS in isolated mouse islets.

## Discussion

The physiological and pathophysiological implications of β-cell production of NO in GSIS are still unresolved and controversial. Many disparate results on the effects of NOS activities hitherto reported can possibly be explained by highly different experimental conditions and also the use of various β-cell-derived tumor cell lines instead of intact islets. Moreover, different culture and/or preincubation conditions for the islets seem to highly influence the results [6,29]. A technical and fundamental observation in this regard is the present finding that islet culture or preincubation at glucose levels above basal induced a large and long-lasting generation of iNOS-derived NO thus interfering with the evaluation of downstream experiments. Moreover, as shown here, murine and also human islets cultured in conventional RPMI 1640 medium containing 11.1 mM glucose induced a large iNOS expression being still evident after washing and preincubation at basal glucose for 30 min. Thus, culturing or preincubating at glucose levels above basal will confound downstream NO measurements in relation to the secretion of insulin in subsequent GSIS experiments. These data are also in line with previous findings in islets from the young hyperglycemic, diabetic GK rat, where an abnormally increased level of iNOS activity in freshly isolated islets persisted even after a downstream incubation for 2h at basal glucose [7]. Therefore, to obtain the true nNOS activity regulating GSIS simultaneous measurements of nNOS *vs* iNOS activity are necessary.

### Acute effects of nNOS inhibition on GSIS

The present results, obtained in 60 min. experiments, clearly showed that an increase in GSIS is inversely related to nNOS activity. Thus a marked suppression of nNOS activity by appropriate concentrations of the L-arginine based NOS inhibitors L-NAME and L-NMMA, both shown to be useful inhibitors of islet nNOS activity [6,21], resulted in a marked amplification of GSIS. A late and modest increase in iNOS activity [6] did not affect GSIS and is thus only confusing. The present data are in line with our previous observation in perifused rat islets, showing that the first 10 min of GSIS is accompanied by increased nNOS activity but no iNOS activity and that suppression of nNOS activity resulted in an increased insulin response [6]. Moreover, an inhibitory effect of L-NAME on nNOS activity is accomplished also *in vivo* already at 2 min after iv injection of the inhibitor and coincides with a marked stimulation of GSIS [35]. Further, a full dose of an nNOS inhibitor such as L-NAME or L-NMMA has been shown to almost abolish the nadir between first and second phase of GSIS in perifused islets and in the perfused pancreas [6,8,22]. The putative importance of nNOS as part of a negative feedback regulation of GSIS was previously also indicated in isolated islets from the obese *ob/ob* mouse. Islets isolated from such mice, when incubated at high glucose, showed a paradoxical decrease in nNOS activity coincident with an exaggerated insulin release [36]. The *ob/ob* mouse is deficient in the hormone leptin [37]. Leptin has been found to markedly stimulate nNOS activity in islets from these mice coinciding with a marked inhibitory effect on GSIS [36]. Furthermore, islets from *ob/ob* mice did not show the expected increase in GSIS observed in control islets when incubated with the nNOS inhibitor L-NAME [36]. Importantly, and in accordance with these results, it has been shown that islets from obese humans as well as from the obese Zucker fa/fa rat displayed a markedly increased content of catalytically inactive nNOS dimers associated with a highly exaggerated GSIS [22]. Moreover, this increase of GSIS was related to an increased islet content of asymmetric dimethyl-arginine (ADMA), an endogenous inhibitor of nNOS with similar properties as L-NAME and L-NMMA [22]. In line with this, there was no effect on GSIS after adding L-NAME to incubated islets from obese humans or to perfused pancreas from obese rats [22]. These data suggested to us that an insufficient increase in β-cell nNOS activity at high glucose might underlie an inappropriate hypersecretion of insulin. Additionally, it has been shown that GSIS is greatly enhanced in nNOS knockout mice [38]. It should be recalled that many effects exerted by NO in different cell types are often exerted through rapid S-nitrosylation processes [30,31,39]. Hence, the proximity between nNOS and its target protein(s) or other SH-containing molecules might be of high significance, being facilitated here by the close association between nNOS and the secretory granules [13,22]. Notably, a close association has been shown also between insulin secretory granules and NADPH [40], linking this indispensable cofactor for upregulation of nNOS activity intimately to the secretory machinery.

### The PPP, nNOS and NADPH

Since L-NAME as shown here (Fig 2D) induced a strong amplification of GSIS in K^+^ diazoxide-treated islets we found it less likely that the main action of nNOS was directed to the glycolytic and the mitochondrial pathways but rather to the PPP and the glutaredoxin-1 system [25,26]. A high redox state of the cytosolic NADPH/NADP ratio is essential for keeping the glutathione system in the reduced state, and the availability of reduced glutathione (GSH) has since long been suggested as essential for a positive modulation of GSIS in the PPP [41]. This was later on underlined by the finding that NADPH could stimulate rat β-cell exocytosis through activating the cytosolic redox protein glutaredoxin-1, a cofactor of GSH, being preferentially localized to the secretory granules in the β-cell periphery [25,26]. Since cytosolic NADPH is produced early in β-cell glucose metabolism via G-6-PD, and since the generation of nNOS-derived NO is an early event in GSIS [23,42], it is tempting to assume that in the PPP, during glucose stimulation, an overconsumption of G-6-PD-derived NADPH by the glucose-stimulated activity of nNOS leads to a situation, where this increase in nNOS activity and thus NADPH consumption as well the ensuing NO production might interfere with and suppress a putative alternative secretory signal elaborated by the NADPH-glutaredoxin-1 system. Further, as shown here, the insulin response to high glucose was reduced by approximately 50% in the presence of the recognized G-6-PD inhibitor DHEA [24] suggesting that half of the response was executed in the PPP. A similar 50% reduction of GSIS by DHEA was previously reported in rat islets [24]. Moreover, these authors also showed that the expected increase of the ATP/ADP ratio was unaffected by DHEA, and thus DHEA did not influence the triggering signal induced by the glycolytic and mitochondrial metabolism of glucose [24]. Accordingly, as shown here, after inhibition of nNOS activity by L-NAME, GSIS was increased 2-fold but similarly reduced again after adding DHEA. Further, the present data from using D-glyceraldehyde as a metabolic triggering substitute for the glycolytic and mitochondrial glucose metabolism [27,28] together with a basal subtriggering, concentration of glucose, 7 mM, (Fig 1B) showed that this combination resulted in an insulin response of the same magnitude as after high glucose. This response was again markedly suppressed by DHEA suggesting that a significant part of glucose signaling, also at this basal glucose concentration, subtriggering for the glycolytic and the mitochondrial pathways, was transduced through the PPP and thus negatively afflicted by the inhibitor. Moreover, the present data also conform with previous results from rat islets showing that silencing of glutaredoxin-1 reduced GSIS by 46% and total exocytosis by 38% in rat β-cells [25,26]. Interestingly, G-6-PD protein expression is reportedly very low in β-cells, and even a modest decrease in G-6-PD activity is able to increase ROS (reactive oxygen species), and might also lead to increased GSH oxidation and dysregulation of GSIS [24,43]. This can be counteracted by overexpression of G-6-PD [43]. Further, it has also been shown that the activity of G-6-PD can be selectively upregulated in response to glucose, while downstream products of G-6-PD were generally less afflicted [44]. All these data point to the PPP being of great importance for GSIS and thus in turn speak in favor of nNOS being able to serve as a potent regulator of the secretory process in the PPP. In this context, it should be recalled that G-6PD deficiency in patients is reportedly associated with impaired insulin secretion [45]. Although, the experiments with the recognized G-6-PD inhibitor DHEA suggest the PPP being the main target for nNOS, it should be recalled that a certain and minor population of nNOS is not associated with the insulin secretory granules [13,22]. This population is mainly located to the mitochondria and thus might impact the mitochondrial metabolism [13,22]. Further, in addition to nNOS also different other factors are known to impact the PPP and thus the modulation of GSIS [1–3,24,43,44].

### Possible importance of nNOS-associated secretory granules and a local negative feedback mechanism in the PPP

Early observations in isolated islets showed that the levels of both NADPH and GSH were increased after glucose stimulation and that this increase could be reversed after addition of exogenous insulin [41]. Moreover, intracellular addition of NADPH as well as downstream activation of the redox protein glutaredoxin-1 enhanced Ca^2+^-induced exocytosis [25,26]. Hence, there is reason to believe that the glucose-induced increase in G-6-PD-derived NADPH in the PPP starts a sequence of events that initiates an alternative pathway for insulin exocytosis. Our present data might thus suggest that this alternative pathway for GSIS can be negatively afflicted by upregulation of nNOS activity through its competition for and utilization of the G-6-PD-derived NADPH. The association of nNOS with a population of secretory granules [13,22] as well as the proximity to glutaredoxin-1 might constitute a morphological basis for distinct nNOS-associated secretory granule populations elaborating such an alternative pathway for GSIS [25,26]. The functional existence of certain nNOS-associated compartments seems feasible, since the large content of cellular proteins capable of being nitrosylated must be avoided. It should be recalled that glucokinase controls the conversion of glucose to G-6-P and that dissociation of glucokinase from the granule-associated nNOS is a step prior to G-6-PD activation [46]. Hence, a rapid S-nitrosylation process by nNOS to activate glucokinase [39,46] might constitute a very early event preceding the point where the increased levels of nNOS activity starts to negatively afflict the PPP activity. It has been shown previously that this early glucose stimulation of the granule-bound nNOS to S-nitrosylate and activate glucokinase is followed by nNOS-derived S-nitrosylation of different targets for granule fusion and exocytosis [39]. Importantly, an increasing glucose concentration in the nNOS-associated compartments of the PPP might then lead to a negative feedback effect in these compartments exerted by the increasing amount of accumulating insulin molecules at the exocytotic sites of the nNOS-associated granules. This process might be regarded as local rather than global and thus probably restricted to the nNOS granules in the PPP. Moreover, previous studies [26] have suggested that local glutareduxin-1-catalyzed reaction might be involved in insulin exocytosis in specific compartments. As we have suggested previously [47], such a negative feedback is most conceivably exerted through insulin receptors mediating activation of nNOS, which then starts to utilize NADPH in the PPP. Thus, by measuring C-peptide secretion from isolated islets we found that high concentrations of added insulin induced an inhibitory effect on GSIS coinciding with an increased nNOS activity. Intermediate insulin concentrations had no effect, while low insulin concentrations increased the secretory activity without changing the nNOS activity [47]. The exact nature of the insulin receptors conveying inhibition vs stimulation of insulin secretion is currently unclear [48]. A further suggestion for the implication of nNOS activity in the PPP is the present observation showing that the inhibition of this nNOS-dependent part of GSIS still could be relieved by L-NAME in K^+^-diazoxide-treated islets, consequently operating largely independent of depolarization events and conceivably acting in addition to the ATP-induced triggering events. This is in contrast to other nutrient-based secretagouges such as *e*.*g*. leucine or α-ketoisocaproic acid (KIC) the signaling of which is known to be initiated directly in the tricarboxylic acid cycle. The effect of KIC is not influenced by L-NAME in depolarized islets and thus bypassing the PPP [49]. Conceivably, it is glucose signaling via NADPH in the PPP that is afflicted by nNOS and not the exocytotic mechanisms per se.

### Is an extracellular approach of NO during GSIS of relevance?

Being a freely permeable gas NO is able to diffuse both in and out of the β-cell and eventually reach the surface of neighboring cells. However, the highly reactive NO is easily trapped within the microenvironment of the β-cell by *e*.*g*. S-nitrosylation and/or tyrosine nitration [30,31]. Hence the role of NO in GSIS is complex and has been evaluated not only by studying the effects on its generation within the β-cell but also, extensively, by means of exogenous addition of NO donors or NO gas to isolated islets and β-cells, perfused pancreas, or tumor β-cell lines, all experiments giving highly contradictory results [6,29]. The effects of NO donors depend not only on their chemical reactivity and kinetics of NO release, but also on their ability to cross cell membranes and eventually exert their action intracellularly. Hence, the use of exogenous sources of NO might not properly recapitulate the action of NO produced in the intra-β-cell milieu. Besides, different NO donors generate different bioactive byproducts following decomposition and metabolism [50]. We show here that exogenously added pure NO gas to islets incubated at high glucose significantly amplified GSIS at a narrow concentration range of NO, whereas then increasing concentrations of the gas gradually inhibited the insulin releasing process down to almost a total suppression at a saturated NO concentration. Similarly, several studies have shown by measuring effects of NO donors on the pattern of basal and glucose-induced Ca^2+^-oscillations and Ca^2+^ redistribution in the β-cells as well as on the influence on K^+^_ATP_-channels, that a low concentration of NO was stimulatory while a high concentration was inhibitory to β-cell activity [51]. The stimulatory effect by NO on GSIS in the present study was abolished in K^+^-diazoxide-treated islets and the inhibitory effect was even more pronounced suggesting that the inhibitory effect was independent of depolarization-triggering events, and thus most likely inducing its inhibitory action through suppressing the alternative secretory pathway executed in the PPP by G-6-PD-NADPH-glutaredoxin-1 signaling. Importantly, as seen here at a concentration of 1 mM glucose, being far below the threshold for GSIS, the positive modulatory effect of low NO on insulin secretion relative to controls was still more pronounced than at high glucose. Moreover, it was abolished by K^+^-diazoxide treatment, while the inhibitory effect at a high concentration of NO was still evident. Hence, the negative modulation of intra-β-cell activation of nNOS on GSIS through its restraining action on the activity of the G-6-PD-NADPH-glutaredoxin-1 system might be separated from a positive extracellular action on the β-cell plasma membrane by a low NO concentration. Such a notion was still more apparent after blocking the NO-induced stimulation of GSIS by the application of SITS thus converting it to a concentration-dependent suppression even far below the control level. Evidently, the inhibitory action of NOS activity on GSIS in SITS-blocked islets became even more pronounced by an increased inflow of nontrapped extracellular NO. Further, with regards to the concentration-response curve for NO-stimulated insulin secretion at 20 mM glucose it should be remarked that the strong inhibition of insulin secretion revealed at 200–2000 μM of NO might include a slight impact of hypoxia. On the other hand, it might be worth mentioning that dietary nitrate fuels as well as nitrate administration have revealed a nitrate-nitrite-NO pathway that can affect the β-cell in a positive way by stimulating basal insulin secretion via an extracellular mode of action [52]. Thus, low levels of extracellular NO might enhance β-cell secretion and survival [52,53], while high and sustained levels, mainly emanating from immunologically induced iNOS activity and often delivered by invading inflammatory cells, since long have been known to bring about destructive effects on the β-cells [9,10].

### Comparison with carbon monoxide (CO)

In contrast to the dual action of exogenous NO on the insulin secretory machinery the comparative experiment with CO showed here that both low and high concentrations of exogenous CO amplified GSIS. This is in accordance with our previous results [19] showing an increased GSIS after adding increasing amounts of the HO-substrate hemin to incubated islets at high glucose, and thus underlining that endogenous CO might be an important amplifier of GSIS. Notably, the insulin secretory concentration-response curve after exogenous CO was clearly bell-shaped, suggesting that extremely high CO concentrations might induce adverse effects as also shown in other tissues [54]. It cannot be excluded that these negative effects also might include the presence of a slight hypoxia as previously discussed for high concentrations of NO. Moreover, in the presence of high glucose, addition to isolated islets of a CO concentration of 1 μM, approximately comparable to endogenous CO levels in brain tissue [55], has been shown to induce an amplification of GSIS that is completely inhibited by the specific guanylate cyclase inhibitor ODQ [19]. In contrast to our findings with exogenous NO, the effects of exogenous CO were abolished in K^+^-diazoxide–treated islets, suggesting membrane depolarization involvement in CO-stimulated GSIS. In this context it is worth noting that it has been shown that NO as well as CO can act as special messengers propagating Ca^2+^ signals coordinating β-cell rhythmicity [56,57], and that nNOS- containing nerves as well as ganglion cells containing nNOS or HO-2 have been localized to islet tissue [21,34,57].

### Cyclic nucleotides

Finally, several previous observations have indicated that NO gas or NO donors are able to stimulate islet cyclic GMP levels conceivably through the activation of soluble guanylate cyclase [29]. Interestingly, in the present study, measurement of islet content of cyclic GMP and cyclic AMP during GSIS at high glucose showed no effect on either cyclic GMP or cyclic AMP levels after exposure of the islets to a low concentration of exogenous NO. Moreover, a saturated concentration of exogenous NO displayed a slight suppression of cyclic GMP and a strong suppression of cyclic AMP. In contrast, we found here a significant increase in cyclic GMP both at a low and a high concentration of exogenous CO, and a modest increase in cyclic AMP at a high concentration of CO. These data thus suggest that it might be CO and not NO that preferentially stimulates cyclic GMP during GSIS. Hence, during GSIS and exogenous stimulation by NO or CO there seems to be a complex interaction between NO-CO and the cyclic nucleotide messengers.

## Conclusion

**Fig 5:**
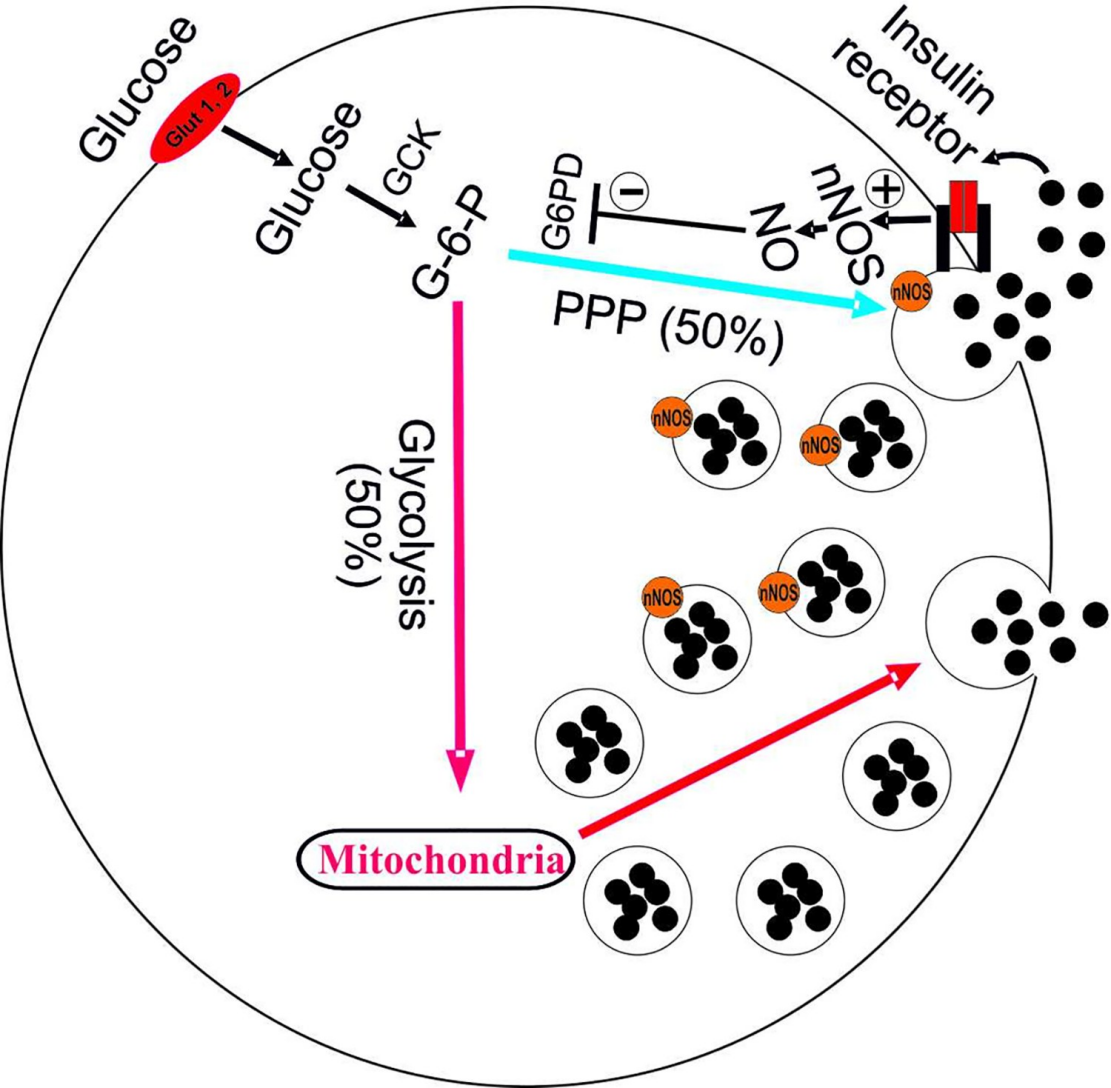
Simplified scheme illustrating the inhibitory effect of nNOS on the PPP during the initiation of GSIS. 1) A marked increase in glucose concentration results in a large accumulation of the released insulin at the exocytotic sites of nNOS-associated insulin granules (denoted as orange circles on insulin granules) in the PPP. The PPP is denoted by blue arrow and the glycolytic and mitochondrial pathways are denoted by red arrows. 2) The accumulated insulin levels stimulate nNOS activity through insulin receptor signalling. 3) The increase in nNOS activity including NADPH consumption inhibits G-6-PD activity in the PPP and thus interferes with and induces an inhibitory action in the PPP part of GSIS. 4) Extended inhibition of the PPP might lead to overload and derangement of the glycolytic and mitochondrial pathways. *Abbreviations*: Glucose transporter 1 or 2 (Glut 1, 2); Glucokinase (GCK), Glucose-6 phosphate (G-6-P), Glucose-6-phosphate dehydrogenase (G-6-PD), Pentose phosphate pathway (PPP). Neuronal nitric oxide synthase (nNOS).

Although an extracellular approach of NO might have some influence on GSIS, it is evident that modulation of the β-cell nNOS activity is of high significance. Here, the present data speak in favor of islet nNOS being an important negative regulator of GSIS through its utilization of NADPH and its production of NO in the PPP and thereby inhibiting the secretory activity induced by the G-6-PD-NADPH-glutaredoxin-1 system preferentially located to certain compartments of nNOS-associated secretory granules. The activation of nNOS in GSIS is most likely transduced by a negative feedback mechanism [47] excited by the accumulating insulin molecules at the exocytotic sites of these nNOS-associated secretory granules in the PPP. Notably, pronounced inhibitory effects on the PPP might in turn have negative implications also for the glycolytic and mitochondrial pathways and thus lead to further derangements of GSIS [43–45,57–60]. In addition, a direct negative effect by nNOS on mitochondrial metabolism cannot be excluded [13,22]. The alternative pathway along the PPP seems initially to involve as much as approximately 50% of the total GSIS. Derangement of this pathway by extended high glucose levels leads to increased nNOS activity followed with time by elevated iNOS activity as reflected in *e*.*g*. the diabetic GK rat as well as in human T2D being subjected to a persistent high glucose [7,8,11]. Moreover, increased expression levels of both nNOS and iNOS have been found in human postmortem T2D islets [15]. Based on the present results and previously reported findings [6–8,11,14–16,19–21,33–36,47,49,57], a very simplified schematic image of our hypothesis concerning the involvement of nNOS-associated granules in the regulation of GSIS is suggested (Fig 5). Moreover, inhibition of G-6-PD in the PPP by an inappropriate and marked increase in nNOS activity might result in an overloaded activity of the glycolytic and mitochondrial pathways that consequently would result in a deranged GSIS. Hence, therapeutic means in the future might include new pharmacological strategies modifying islet nNOS activity in T2D.

## Supporting information

S1 File (XLSX)

## References

[1] Straub SG, Sharp GW (2002) Glucose-stimulated signaling pathways in biphasic insulin secretion. Diabetes Metab Res Rev 18: 451–463. doi: 10.1002/dmrr.329 12469359

[2] Prentki M, Matschinsky FM (1987) Ca2+, cAMP, and phospholipid-derived messengers in coupling mechanisms of insulin secretion. Physiol Rev 67: 1185–1248. doi: 10.1152/physrev.1987.67.4.1185 2825225

[3] Ishihara H (2022) Metabolism-secretion coupling in glucose-stimulated insulin secretion. Diabetol Int 13: 463–470. doi: 10.1007/s13340-022-00576-z 35693987 PMC9174369

[4] Knowles RG, Moncada S (1994) Nitric oxide synthases in mammals. Biochem J 298 (Pt 2): 249–258. doi: 10.1042/bj2980249 7510950 PMC1137932

[5] Alderton WK, Cooper CE, Knowles RG (2001) Nitric oxide synthases: structure, function and inhibition. Biochem J 357: 593–615. doi: 10.1042/0264-6021:3570593 11463332 PMC1221991

[6] Henningsson R, Salehi A, Lundquist I (2002) Role of nitric oxide synthase isoforms in glucose-stimulated insulin release. Am J Physiol Cell Physiol 283: C296–304. doi: 10.1152/ajpcell.00537.2001 12055099

[7] Salehi A, Meidute Abaraviciene S, Jimenez-Feltstrom J, Ostenson CG, Efendic S, Lundquist I (2008) Excessive islet NO generation in type 2 diabetic GK rats coincides with abnormal hormone secretion and is counteracted by GLP-1. PLoS One 3: e2165. doi: 10.1371/journal.pone.0002165 18478125 PMC2367446

[8] Mosen H, Ostenson CG, Lundquist I, Alm P, Henningsson R, Jimenez-Feltstrom J, et al. (2008) Impaired glucose-stimulated insulin secretion in the GK rat is associated with abnormalities in islet nitric oxide production. Regul Pept 151: 139–146. doi: 10.1016/j.regpep.2008.07.002 18662725

[9] Eizirik DL, Flodstrom M, Karlsen AE, Welsh N (1996) The harmony of the spheres: inducible nitric oxide synthase and related genes in pancreatic beta cells. Diabetologia 39: 875–890. doi: 10.1007/BF00403906 8858209

[10] Corbett JA, Sweetland MA, Wang JL, Lancaster JR, Jr., McDaniel ML(1993) Nitric oxide mediates cytokine-induced inhibition of insulin secretion by human islets of Langerhans. Proc Natl Acad Sci U S A 90: 1731–1735. doi: 10.1073/pnas.90.5.1731 8383325 PMC45953

[11] Muhammed SJ, Lundquist I, Salehi A (2012) Pancreatic beta-cell dysfunction, expression of iNOS and the effect of phosphodiesterase inhibitors in human pancreatic islets of type 2 diabetes. Diabetes Obes Metab 14: 1010–1019. 22687049 10.1111/j.1463-1326.2012.01632.x

[12] Defronzo RA (2009) Banting Lecture. From the triumvirate to the ominous octet: a new paradigm for the treatment of type 2 diabetes mellitus. Diabetes 58: 773–795. doi: 10.2337/db09-9028 19336687 PMC2661582

[13] Lajoix AD, Reggio H, Chardes T, Peraldi-Roux S, Tribillac F, Roye M, et al. (2001) A neuronal isoform of nitric oxide synthase expressed in pancreatic beta-cells controls insulin secretion. Diabetes 50: 1311–1323. doi: 10.2337/diabetes.50.6.1311 11375331

[14] Jimenez-Feltstrom J, Lundquist I, Salehi A (2005) Glucose stimulates the expression and activities of nitric oxide synthases in incubated rat islets: an effect counteracted by GLP-1 through the cyclic AMP/PKA pathway. Cell Tissue Res 319: 221–230. doi: 10.1007/s00441-004-1013-4 15558323

[15] Mohammed Al-Amily I, Lundquist I, Salehi A (2019) Expression levels of enzymes generating NO and CO in islets of murine and human diabetes. Biochem Biophys Res Commun 520: 473–478. doi: 10.1016/j.bbrc.2019.10.055 31607476

[16] Salehi A, Carlberg M, Henningson R, Lundquist I (1996) Islet constitutive nitric oxide synthase: biochemical determination and regulatory function. Am J Physiol 270: C1634–1641. doi: 10.1152/ajpcell.1996.270.6.C1634 8764145

[17] Al-Amily IM, Duner P, Groop L, Salehi A (2019) The functional impact of G protein-coupled receptor 142 (Gpr142) on pancreatic beta-cell in rodent. Pflugers Arch 471: 633–645. 30767071 10.1007/s00424-019-02262-7 PMC6435787

[18] Bradford MM (1976) A rapid and sensitive method for the quantitation of microgram quantities of protein utilizing the principle of protein-dye binding. Anal Biochem 72: 248–254. doi: 10.1016/0003-2697(76)90527-3 942051

[19] Henningsson R, Alm P, Ekstrom P, Lundquist I (1999) Heme oxygenase and carbon monoxide: regulatory roles in islet hormone release: a biochemical, immunohistochemical, and confocal microscopic study. Diabetes 48: 66–76. doi: 10.2337/diabetes.48.1.66 9892224

[20] Mosen H, Salehi A, Henningsson R, Lundquist I (2006) Nitric oxide inhibits, and carbon monoxide activates, islet acid alpha-glucoside hydrolase activities in parallel with glucose-stimulated insulin secretion. J Endocrinol 190: 681–693. doi: 10.1677/joe.1.06890 17003269

[21] Henningsson R, Alm P, Lindstrom E, Lundquist I (2000) Chronic blockade of NO synthase paradoxically increases islet NO production and modulates islet hormone release. Am J Physiol Endocrinol Metab 279: E95–E107. doi: 10.1152/ajpendo.2000.279.1.E95 10893328

[22] Mezghenna K, Pomies P, Chalancon A, Castex F, Leroy J, Niclauss N, et al. (2011) Increased neuronal nitric oxide synthase dimerisation is involved in rat and human pancreatic beta cell hyperactivity in obesity. Diabetologia 54: 2856–2866. doi: 10.1007/s00125-011-2264-8 21847584

[23] Nunemaker CS, Buerk DG, Zhang M, Satin LS (2007) Glucose-induced release of nitric oxide from mouse pancreatic islets as detected with nitric oxide-selective glass microelectrodes. Am J Physiol Endocrinol Metab 292: E907–912. doi: 10.1152/ajpendo.00518.2006 17122087

[24] Spegel P, Sharoyko VV, Goehring I, Danielsson AP, Malmgren S, Nagorny CL, et al. (2013) Time-resolved metabolomics analysis of beta-cells implicates the pentose phosphate pathway in the control of insulin release. Biochem J 450: 595–605. 23282133 10.1042/BJ20121349

[25] Ivarsson R, Quintens R, Dejonghe S, Tsukamoto K, in ’t Veld P, Renstrom E, et al. (2005) Redox control of exocytosis: regulatory role of NADPH, thioredoxin, and glutaredoxin. Diabetes 54: 2132–2142. doi: 10.2337/diabetes.54.7.2132 15983215

[26] Reinbothe TM, Ivarsson R, Li DQ, Niazi O, Jing X, Zhang E, et al. (2009) Glutaredoxin-1 mediates NADPH-dependent stimulation of calcium-dependent insulin secretion. Mol Endocrinol 23: 893–900. doi: 10.1210/me.2008-0306 19299446 PMC5419284

[27] Hellman B, Idahl LA, Lernmark A, Sehlin J, Taljedal IB (1974) The pancreatic beta-cell recognition of insulin secretagogues. Comparisons of glucose with glyceraldehyde isomers and dihydroxyacetone. Arch Biochem Biophys 162: 448–457. doi: 10.1016/0003-9861(74)90204-5 4210076

[28] Ashcroft SJ, Weerasinghe LC, Randle PJ (1973) Interrelationship of islet metabolism, adenosine triphosphate content and insulin release. Biochem J 132: 223–231. doi: 10.1042/bj1320223 4199014 PMC1177581

[29] Gheibi S, Ghasemi A (2020) Insulin secretion: The nitric oxide controversy. EXCLI J 19: 1227–1245. doi: 10.17179/excli2020-2711 33088259 PMC7573190

[30] Jaffrey SR, Erdjument-Bromage H, Ferris CD, Tempst P, Snyder SH (2001) Protein S-nitrosylation: a physiological signal for neuronal nitric oxide. Nat Cell Biol 3: 193–197. doi: 10.1038/35055104 11175752

[31] Foster MW, Hess DT, Stamler JS (2009) Protein S-nitrosylation in health and disease: a current perspective. Trends Mol Med 15: 391–404. doi: 10.1016/j.molmed.2009.06.007 19726230 PMC3106339

[32] Hellman B, Idahl L-Å, Lernmark Å, Sehlin J, Täljedal I-B (1974) Membrane sulphydryl groups in pancreatic β-cell recognition of insulin secretagogues; Malaisse WJ PJ, editor: pp. 65–78. Excerpta Medica Int. Congr. Ser 312, 500 p.

[33] Henningsson R, Alm P, Lundquist I (2001) Evaluation of islet heme oxygenase-CO and nitric oxide synthase-NO pathways during acute endotoxemia. Am J Physiol Cell Physiol 280: C1242–1254. doi: 10.1152/ajpcell.2001.280.5.C1242 11287338

[34] Alm P, Ekstrom P, Henningsson R, Lundquist I (1999) Morphological evidence for the existence of nitric oxide and carbon monoxide pathways in the rat islets of Langerhans: an immunocytochemical and confocal microscopical study. Diabetologia 42: 978–986. doi: 10.1007/s001250051256 10491758

[35] Akesson B, Henningsson R, Salehi A, Lundquist I (1999) Islet constitutive nitric oxide synthase and glucose regulation of insulin release in mice. J Endocrinol 163: 39–48. doi: 10.1677/joe.0.1630039 10495405

[36] Jimenez-Feltstrom J, Salehi A, Meidute Abaraviciene S, Henningsson R, Lundquist I (2011) Abnormally decreased NO and augmented CO production in islets of the leptin-deficient ob/ob mouse might contribute to explain hyperinsulinemia and islet survival in leptin-resistant type 2 obese diabetes. Regul Pept 170: 43–51. doi: 10.1016/j.regpep.2011.04.011 21620903

[37] Seufert J (2004) Leptin effects on pancreatic beta-cell gene expression and function. Diabetes 53 Suppl 1: S152–158. doi: 10.2337/diabetes.53.2007.s152 14749281

[38] Hao M, Head WS, Gunawardana SC, Hasty AH, Piston DW (2007) Direct effect of cholesterol on insulin secretion: a novel mechanism for pancreatic beta-cell dysfunction. Diabetes 56: 2328–2338. doi: 10.2337/db07-0056 17575085

[39] Wiseman DA, Thurmond DC (2012) The good and bad effects of cysteine S-nitrosylation and tyrosine nitration upon insulin exocytosis: a balancing act. Curr Diabetes Rev 8: 303–315. doi: 10.2174/157339912800840514 22587517 PMC3571098

[40] Watkins DT, Moore M (1977) Uptake of NADPH by islet secretion granule membranes. Endocrinology 100: 1461–1467. doi: 10.1210/endo-100-5-1461 14821

[41] Ammon HP, Grimm A, Lutz S, Wagner-Teschner D, Handel M, Hagenloh I (1980) Islet glutathione and insulin release. Diabetes 29: 830–834. doi: 10.2337/diacare.20.10.830 7002664

[42] Nakada S, Ishikawa T, Yamamoto Y, Kaneko Y, Nakayama K (2003) Constitutive nitric oxide synthases in rat pancreatic islets: direct imaging of glucose-induced nitric oxide production in beta-cells. Pflugers Arch 447: 305–311. doi: 10.1007/s00424-003-1176-y 14564523

[43] Zhang Z, Liew CW, Handy DE, Zhang Y, Leopold JA, Hu J, et al. (2010) High glucose inhibits glucose-6-phosphate dehydrogenase, leading to increased oxidative stress and beta-cell apoptosis. FASEB J 24: 1497–1505. doi: 10.1096/fj.09-136572 20032314 PMC2879949

[44] Huang M, Joseph JW (2014) Assessment of the metabolic pathways associated with glucose-stimulated biphasic insulin secretion. Endocrinology 155: 1653–1666. doi: 10.1210/en.2013-1805 24564396

[45] Stanton RC (2012) Glucose-6-phosphate dehydrogenase, NADPH, and cell survival. IUBMB Life 64: 362–369. doi: 10.1002/iub.1017 22431005 PMC3325335

[46] Markwardt ML, Seckinger KM, Rizzo MA (2016) Regulation of Glucokinase by Intracellular Calcium Levels in Pancreatic beta Cells. J Biol Chem 291: 3000–3009. 26698632 10.1074/jbc.M115.692160 PMC4742761

[47] Jimenez-Feltstrom J, Lundquist I, Obermuller S, Salehi A (2004) Insulin feedback actions: complex effects involving isoforms of islet nitric oxide synthase. Regul Pept 122: 109–118. doi: 10.1016/j.regpep.2004.06.004 15380928

[48] Rachdaoui N (2020) Insulin: The Friend and the Foe in the Development of Type 2 Diabetes Mellitus. Int J Mol Sci 21: 1770. doi: 10.3390/ijms21051770 32150819 PMC7084909

[49] Salehi A, Parandeh F, Lundquist I (1998) Signal transduction in islet hormone release: interaction of nitric oxide with basal and nutrient-induced hormone responses. Cell Signal 10: 645–651. doi: 10.1016/s0898-6568(98)00005-9 9794246

[50] Feelisch M (1998) The use of nitric oxide donors in pharmacological studies. Naunyn Schmiedebergs Arch Pharmacol 358: 113–122. doi: 10.1007/pl00005231 9721012

[51] Kurohane Kaneko Y, Ishikawa T (2013) Dual role of nitric oxide in pancreatic beta-cells. J Pharmacol Sci 123: 295–300. 24285083 10.1254/jphs.13r10cp

[52] Nystrom T, Ortsater H, Huang Z, Zhang F, Larsen FJ, Weitzberg E, et al. (2012) Inorganic nitrite stimulates pancreatic islet blood flow and insulin secretion. Free Radic Biol Med 53: 1017–1023. doi: 10.1016/j.freeradbiomed.2012.06.031 22750508

[53] Bedoya FJ, Salguero-Aranda C, Cahuana GM, Tapia-Limonchi R, Soria B, Tejedo JR (2012) Regulation of pancreatic beta-cell survival by nitric oxide: clinical relevance. Islets 4: 108–118. 22614339 10.4161/isl.19822

[54] Wu L, Wang R (2005) Carbon monoxide: endogenous production, physiological functions, and pharmacological applications. Pharmacol Rev 57: 585–630. doi: 10.1124/pr.57.4.3 16382109

[55] Ingi T, Cheng J, Ronnett GV (1996) Carbon monoxide: an endogenous modulator of the nitric oxide-cyclic GMP signaling system. Neuron 16: 835–842. doi: 10.1016/s0896-6273(00)80103-8 8608001

[56] Grapengiesser E, Gylfe E, Dansk H, Hellman B (2001) Nitric oxide induces synchronous Ca2+ transients in pancreatic beta cells lacking contact. Pancreas 23: 387–392. doi: 10.1097/00006676-200111000-00009 11668208

[57] Lundquist I, Alm P, Salehi A, Henningsson R, Grapengiesser E, Hellman B (2003) Carbon monoxide stimulates insulin release and propagates Ca2+ signals between pancreatic beta-cells. Am J Physiol Endocrinol Metab 285: E1055–1063. doi: 10.1152/ajpendo.00498.2002 14534076

[58] Rebelato E, Abdulkader F, Curi R, Carpinelli AR (2011) Control of the intracellular redox state by glucose participates in the insulin secretion mechanism. PLoS One 6: e24507. doi: 10.1371/journal.pone.0024507 21909396 PMC3164208

[59] Zhang E, Mohammed Al-Amily I, Mohammed S, Luan C, Asplund O, Ahmed M, et al. (2019) Preserving Insulin Secretion in Diabetes by Inhibiting VDAC1 Overexpression and Surface Translocation in beta Cells. Cell Metab 29: 64–77 e66. 30293774 10.1016/j.cmet.2018.09.008 PMC6331340

[60] Chareyron I, Christen S, Moco S, Valsesia A, Lassueur S, Dayon L, et al. (2020) Augmented mitochondrial energy metabolism is an early response to chronic glucose stress in human pancreatic beta cells. Diabetologia 63: 2628–2640. doi: 10.1007/s00125-020-05275-5 32960311 PMC7641954

